# The perception of gender in two allegedly sex-specific body odor compounds MSH and HMHA

**DOI:** 10.1038/s41598-025-26457-4

**Published:** 2025-11-27

**Authors:** Camille Ferdenzi, Géraldine Coppin, Christophe Bousquet, Sylvain Delplanque

**Affiliations:** 1 Claude Bernard University Lyon 1, Centre de Recherche en Neurosciences de Lyon CRNL, CNRS UMR5292, INSERM U1028, Bron Cedex, F-69500 France; 2 Department of Psychology, UniDistance Suisse, Brig, Switzerland; 3 Swiss Center for Affective Sciences, University of Geneva, Geneva, Switzerland; 4 Department of Psychology, Centre for the Advanced Study of Collective Behaviour, University of Konstanz, Konstanz, Germany

**Keywords:** Olfaction, Body odor, Mate preferences, Gender differences, Development, Human behaviour, Sensory processing

## Abstract

3-methyl-3-sulfanylhexan-1-ol (MSH) and 3-hydroxy-3-methylhexanoic acid (HMHA) are two allegedly sexually dimorphic compounds present in human sweat. While MSH is more typically found in women, HMHA is more typically found in men. Here we investigated whether it was possible to identify these two compounds as explicitly masculine or feminine. We also investigated whether gender and age differences would suggest a possible role of these compounds in mate preferences. To this end, we analyzed the perceptual ratings of t-shirts impregnated with these odors by 2,716 individuals (62% female) aged between 6 and 90, collected during a one-year museum exhibition. Analyses revealed that only women rated MSH as more feminine than HMHA. However, this effect remained very small and masculinity/femininity ratings were extremely variable among the population. Women also rated both odors as more intense and less pleasant than men. Age-related differences reflected the effect of increasing experience with body odors (familiarity). The loss of olfactory function with age was also reflected by decreased perceived intensity and unpleasantness (for HMHA). Overall, the results are not in favor of a role of MSH and/or HMHA in mate preferences, however they do agree with the known gender and age differences in odor perception.

## Introduction

Human body odor is a complex chemical mixture that conveys biological information about individuals^1^. There is increasing evidence that other individuals are able to detect and process this information. For instance, body odor may provide information about genetic proximity^2,3^, hormonal levels^4–6^, sickness^7^, even personality traits^8^ and emotional states^9,10^, and thus appears to regulate our social interactions^11^. In addition, body odor chemical composition varies with age^12,13^, which leads to different perceptual qualities when participants evaluate samples from different age groups^14,15^. Nevertheless, research on human ability to discriminate the body odor of men and women remains inconclusive.

While some studies have found evidence that humans are able to identify body odor donors’ gender from breath odor^16^ and torso odor^17^, the performances in doing so remained rather low. In a study using axillary odors, almost no rater performed above chance in assigning a gender category. Moreover, in the same study, only 22% to 67% of the samples were correctly assigned to their gender category depending on the trials^18^. In another study only 20% to 32% of European participants (but 64% of Asian participants) were able to correctly categorize t-shirt owners’ gender^17^. Also, intensity and pleasantness of the odors were found to influence gender categorization significantly, with stronger and more unpleasant odors being more likely to be categorized as male than female^18^.

Analytical chemistry approaches provide contrasted conclusions as well. Although the chemical composition of human body odor is not fully known partly due to methodological limitations^19,20^, a few studies have investigated gender differences. Some studies fail to find any difference between male and female chemical profiles^12^. Other studies however suggest that, in spite of the large interindividual variability, male and female body odors may differ^21–23^. A consensus regarding the chemicals concerned is far from being reached though, because studies are rare and methods are heterogeneous. In another study on major constituents of human sweat odor^24^, men and women were found to differ in the ratio of two precursors secreted in the armpit. The first one leads to the formation of MSH (3-methyl-3-sulfanylhexan-1-ol), responsible for the sulfurous onion-like odor of women’s sweat. The second precursor leads to the formation of the carboxylic acid HMHA (3-hydroxy-3-methylhexanoic acid), causing a cheesy rancid odor in men’s sweat. In female samples, MSH precursor was found in larger quantities, and HMHA precursor in smaller quantities on average, than in male samples. Although interindividual variability was huge with significant overlap between men and women, the ratio HMHA precursor / MSH precursor was stable and 3 times higher in men than in women. In addition, men have the potential to produce more HMHA than women because they possess a greater amount of corynebacteria^25^, which are responsible for the formation of HMHA^26^. Finally, few studies have been conducted on HMHA and MSH to investigate how they are perceived. Whether HMHA is perceived differently by men and women remains inconclusive to date^27–29^.

In this experiment, we tested whether the more typically feminine compound MSH and the more typically masculine compound HMHA, were perceived as such in an explicit task. The task consisted in rating the odors on a scale from very masculine to very feminine. It was implemented on a very large sample of men and women of all ages in a museum, over a period of one year. Interindividual differences in gender evaluation of the body odor compounds were investigated, with a particular attention to the gender and age of the raters.

Gender differences in odor perception have been shown frequently^30^. Regarding odors in general, women seem to perform better than men in odor identification and memory tasks^31,32^, and display higher attention to odors^33–35^. They do not necessarily have better detection skills though (e.g.,^27,28^). For social odors in particular, women seem to be more responsive to body odors than men (self-declared^36^; hedonic responses to biological samples^37)^. They also identify family members based on their smell more accurately^38^. The present study investigates body odor compounds that are reported to be sexually dimorphic, a characteristic that may have a particular relevance in mate choice (as in other modalities, such as voices^39^ or faces^40^). A few sexually dimorphic body odor compounds have been studied in the past (androstenes), but their supposed role as sex pheromones has never been shown^41,42^. The reasons leading scientists to focus so much on these molecules alone have been criticized^42^. Therefore, our study will contribute to the development of knowledge of other categories of sexually dimorphic body odor compounds. We hypothesize that gender differences may occur, reflecting either a general superiority of women in odor processing and/or specific odor processing related to a role of the compounds in opposite-sex mate preferences (such as men’s higher pleasantness ratings of the female compound MSH and/or women’s higher pleasantness ratings of the male compound HMHA).

Finally, developmental and ageing effects were also expected in this study. Indeed, it is likely that masculinity/femininity of a sweat odor is acquired through experience, and that such judgments become more accurate after puberty (see^43^ for acquired responses to body odors through childhood and adolescence). If MSH and HMHA are relevant for mate choice, it may also be that the information they convey about gender is relevant only during the sexually active period of life, but not during childhood or in the elderly. Last but not least, an age-related decrease in olfactory abilities^32,44^ due to the senescence of the olfactory system may occur and impact the participant’s olfactory evaluations. Overall, the ability to categorize MSH and HMHA as being respectively female and male in our study is expected to be higher in young adults than in the other age groups.

## Methods

### Participants

Between February 15, 2019 and February 23, 2020, a total of 23,236 individuals visited a museum exhibition about the human sense of smell at the Fondation Claude Verdan Musée de la main UNIL-CHUV (Lausanne, Switzerland). One of the interactive modules in the exhibition aimed to test whether it was possible to tell whether two different body odors were masculine or feminine. 2,836 visitors (1,744 women, Age M = 30.7, SD = 18.3; 1,092 men, Age M = 30.0, SD = 20.3) voluntarily and anonymously took part in this module over a period of 10 months [Note: The data collected during the first two months were not retained because the order of presentation of the compounds was fixed, introducing an obvious experimental bias. The correct collection after modification of the experiment presentation software (randomization of compounds delivery) started on 21 May 2019 and ended on 23 February 2020]. In order to avoid the inclusion of inconsistent data in the analyses, we excluded participants who reported being older than 90 years (44 excluded), those not able to smell MSH or HMHA (67 excluded), and those whose answers were the minimum or maximum scores on all questions (9 excluded). Analyses were performed on a final database of 2,716 individuals (1,685 females). The distribution of participants by age group and gender is presented in Table 1.

**Table 1:** Characteristics of the participants (number, age in years: mean ± standard deviation), by gender and age groups.

Age group	Women		Men	
	N	Mean age ± SD	N	Mean age ± SD
6 to 9	123	7.6 ± 1.1	105	7.5 ± 1.2
10 to 19	448	14.2 ± 3.0	293	13.7 ± 2.9
20 to 29	292	24.7 ± 2.9	177	24.9 ± 2.7
30 to 39	333	34.4 ± 3.0	197	34.5 ± 3.2
40 to 49	261	43.9 ± 2.8	142	44.2 ± 2.9
50 to 59	122	53.9 ± 2.7	69	53.6 ± 2.6
60 to 69	80	64.2 ± 3.2	29	64.0 ± 2.8
70 to 90	26	73.8 ± 3.7	19	73.8 ± 4.9

The project has been accepted by the Swiss National Science Foundation (N°CRAGP1_178467) and followed the strict ethical standards set out in The European Code of Conduct for Research Integrity (https://allea.org/portfolio-item/european-code-of-conduct-2023/) and the WMA declaration of Helsinki (https://www.wma.net/policies-post/wma-declaration-of-helsinki/). The need for local ethics committee approval was waived by the Geneva Cantonal Commission on Ethics in human Research (CCER) and the ethic commission of Vaud (Req-2024-01222), because the aim of this completely anonymous study was outside the scope of the Swiss law, defined in Article 2 of the Human Research Act. The study was conducted at the Fondation Claude Verdan Musée de la main in Lausanne, Switzerland, with the museum’s official permission.

### Odorants

1% solutions of MSH (3-methyl-3-sulfanylhexan-1-ol) and HMHA (3-hydroxy-3-methylhexanoic acid) in triacetin, provided by Firmenich, S.A., were diluted respectively 50 times and 5 times in water, leading to a final concentration of 0.02% of MSH and 0.2% of HMHA. Each compound was sprayed (one squeeze, 0.116 ± 0.007 mL) three times a week (Tuesday, Thursday and Saturday), onto a separated piece of cotton cloth (about 10 × 10 cm) cut from a unique plain white T-shirt (renewed between the 10th and 15th of each month). These cloths were then placed at the bottom of two separate glass jars (10 cm diameter, 12 cm height, 188.5 cm^3^), secured to the table and hermetically sealed with a cork. This procedure resulted in two well-perceived odors of fairly similar perceived intensity (as evaluated by a small group of 5 expert colleagues working at Firmenich, S.A.). These relatively high and supraliminal concentrations (HMHA average detection threshold = 12.5 ppm^28^) were chosen to enable conscious perception. It should be borne in mind that these concentrations are most likely different from (higher than) the naturally-occurring concentrations of the compounds, and that this may influence how they are perceived.

### Procedure

**Fig. 1:**
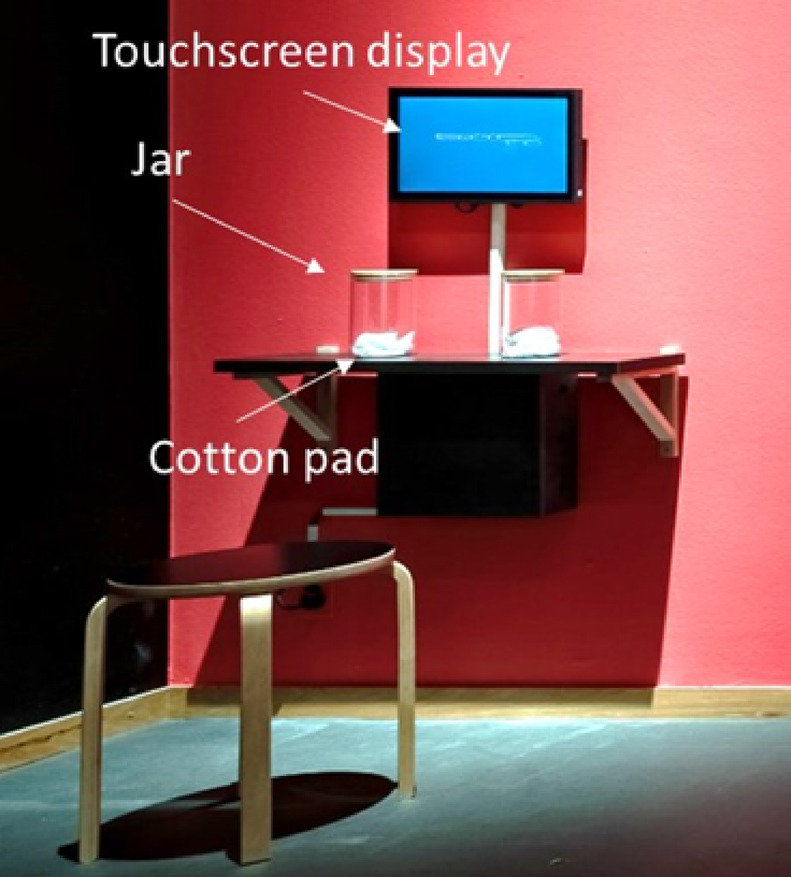
Photograph of the experimental setting in the museum exhibition, including a stool, the two jars and the touch screen to record the participants’ answers.

Visitors to the exhibition who wished to participate freely in this experiment sat on the stool positioned in front of the experimental setup (Fig. 1). They then began the experiment by touching the touch screen on which was written: “Like the smell of sweat… By participating in the following test, you are taking part in a scientific study. Your responses are anonymous and will only be used in this scientific research conducted by the Swiss Center for Affective Sciences of the University of Geneva”. Participation in the test was contingent upon obtaining informed consent. For adult volunteers, informed consent was obtained directly from the participants. For underage volunteers, informed consent was provided by their parents or legal guardians. Participants were invited to provide their gender and age, then to open one of the two jars (random order given by the presentation software), to smell the content, replace the cork and then answer a series of questions using 11-point scales presented on the touch screen. The questions were:

- [Masculinity/Femininity] How would you describe this smell? (from 0 = very masculine to 10 = very feminine);
- [Masculinity/Femininity Certainty] Are you sure of your choice? (0 = not at all, 10 = quite sure);
- [Pleasantness] Do you like this smell? (0 = not at all, 10 = a lot);
- [Intensity] How intense is this smell? (0 = I cannot smell it, 10 = very strong);
- [Familiarity] Do you know this smell? (0 = unknown smell, 10 = very familiar smell);
- [Typicality] For you, does this correspond to typical smell of sweat? (Yes / No).

At the very end of the experiment, the participants could read on the screen: “Thank you for your participation. This scientific research will attempt to answer the following question: Is there a typically masculine or feminine smell of sweat?”.

### Statistical analyses

Data and analyses scripts (performed in R version 4.3.1^40^) can be downloaded at https://osf.io/hdtm7/.

To analyze the gender ratings of the odors MSH and HMHA as well as their modulating factors, we used cumulative link mixed effects models (also known as ordinal mixed effects regressions) in the Bayesian framework with the brms R package^46^. The response variable was the Masculinity/Femininity rating of the odor, an ordinal factor (a Likert-scale) that could take one of eleven values between 0 and 10. The fixed part of the model contained the participants’ socio-demographic characteristics (Rater Age and Rater Gender), the Compound presented (MSH or HMHA) and its interactions with Rater Age and Rater Gender. It also entailed the participant’s other odor ratings (Pleasantness, Intensity, Familiarity and Typicality). Subject’s ID (allowing for individual differences in response to the compound) and compound Presentation Order (first or second) were included as random factors in the model. Additionally, we added a random slope for Compound (MSH or HMHA) to allow for interindividual variation in response to compounds. To facilitate interpretation of the coefficients, all numerical variables (Age, Pleasantness, Intensity and Familiarity) were scaled. As the relationship between Intensity of the odor and its Masculinity/Femininity score was non-linear, we added a quadratic term for Intensity. The binary predictors (Gender, Compound and Typicality of the odor) were treated as factors, with two levels each: Women and Men for Gender (with Women being the reference level), HMHA and MSH for Compound (with HMHA being the reference level), Not typical and Typical for Typicality of the odor (with Not typical being the reference level). As we used a Bayesian framework, we compared models using the Leave-One-Out Information Criterion (looIC), with lower values indicating better fit^47^. The looIC of the full model with a quadratic term for Intensity (looIC = 24308.8) was much lower than the looIC of the full model with a linear relationship for Intensity (looIC = 24369.5), supporting the choice of the quadratic term. The full model can be read as follows:

$$\begin{aligned}\: Masculinity/Femininity\sim RaterGender + RaterAge + Compound + \\ RaterGender \times Compound + Pleasantness + Intensity + Intensity^{2} + \\ Typicality + (1 + Compound|SubjectID) + (1|PresentationOrder)\end{aligned}$$

Besides the full model described above, we also ran a reduced model excluding all variables for which the direction of the effect was not highly certain. It means excluding the variables whose posterior distribution is for at least 10% going in the opposite direction to the median. This is a common practice to avoid overfitting^48^. The reduced model can be read as follows:

$$\begin{aligned}\:Masculinity/Femininity\sim RaterGender + RaterAge + Compound + \\ RaterGender\times Compound + Pleasantness + Intensity + Intensity^{2} + Typicality +\\ (1 + Compound|SubjectID) +(1|PresentationOrder)\end{aligned}$$

We also conducted analyses for the determinants of four other assessments of the odor: its pleasantness, familiarity, intensity, and the certainty of the participant’s Masculinity/Femininity judgment. Pleasantness, Familiarity and Certainty of Masculinity/Femininity rating were each measured on a 11-point Likert scale and were treated as ordinal response variables. Intensity was also originally measured on a 11-point Likert scale. However, our inclusion criteria removed the 67 cases in which participants reported an intensity of 0, i.e., they were not able to smell this odor. Therefore, intensity was treated as a 10-point Likert scale. We ran four separate cumulative link mixed effects models. The same random structure as earlier was applied. We included Rater Gender, Rater Age, Compound, as well as the interactions Rater Gender × Compound, Rater Age × Compound, and Rater Age × Rater Gender × Compound as fixed effects. Two additional elements were included in the model concerning the certainty of Masculinity/Femininity judgment: Gender-strength and Gender-strength × Compound interaction. Gender strength is the result of the transformation of the Masculinity/Femininity scale (ranging from 0 to 10) into a scale ranging from 0 to 5. Zero means gender-neutral odor (i.e., 5 on the original scale) and 5 means highly gendered odor (i.e., either 0/very masculine or 10/very feminine on the original scale). Four reduced models were derived from these four full models by removing all variables for which the direction of the effect was not highly certain (associated probability *p* < 0.90).

For all models, we ran 4 Markov chains in parallel, each with 6,000 iterations, of which the first 2,000 were treated as warmup and were therefore not used for inference. Note that, for all models, when factors were involved in significant interactions, main effects of these factors were not interpreted. We report results from the reduced model within the Bayesian framework by presenting the posterior median slope, its associated Median Absolute Deviation (MAD), the 90% Bayesian Credible Interval (90% CI), the proportion of support for positive or negative effects (p_+_ or p_-_, respectively), as well as derived median and MAD estimates of Cohen’s d for the fixed effects^48^. Even though they come from different frameworks, there is typically a linear relationship between the p-values of the frequentist approach and the p_+_ or p_-_ of the Bayesian approach. Thresholds of 0.05, 0.01 and 0.001 for p-values correspond to thresholds of 0.95, 0.99 and 0.999 for p_+_ or p_-_^49^. Note that when the whole posterior distribution for one parameter is of the same sign, then p_+_ or p_-_ = 1. General guidelines for interpreting Cohen’s *d* are: very large effect when *d* >1.3, large when *d* >0.8, medium when *d* >0.5 and small effect when *d* >0.2^50,51^.

## Results

### Masculinity/Femininity of MSH and HMHA

**Fig. 2:**
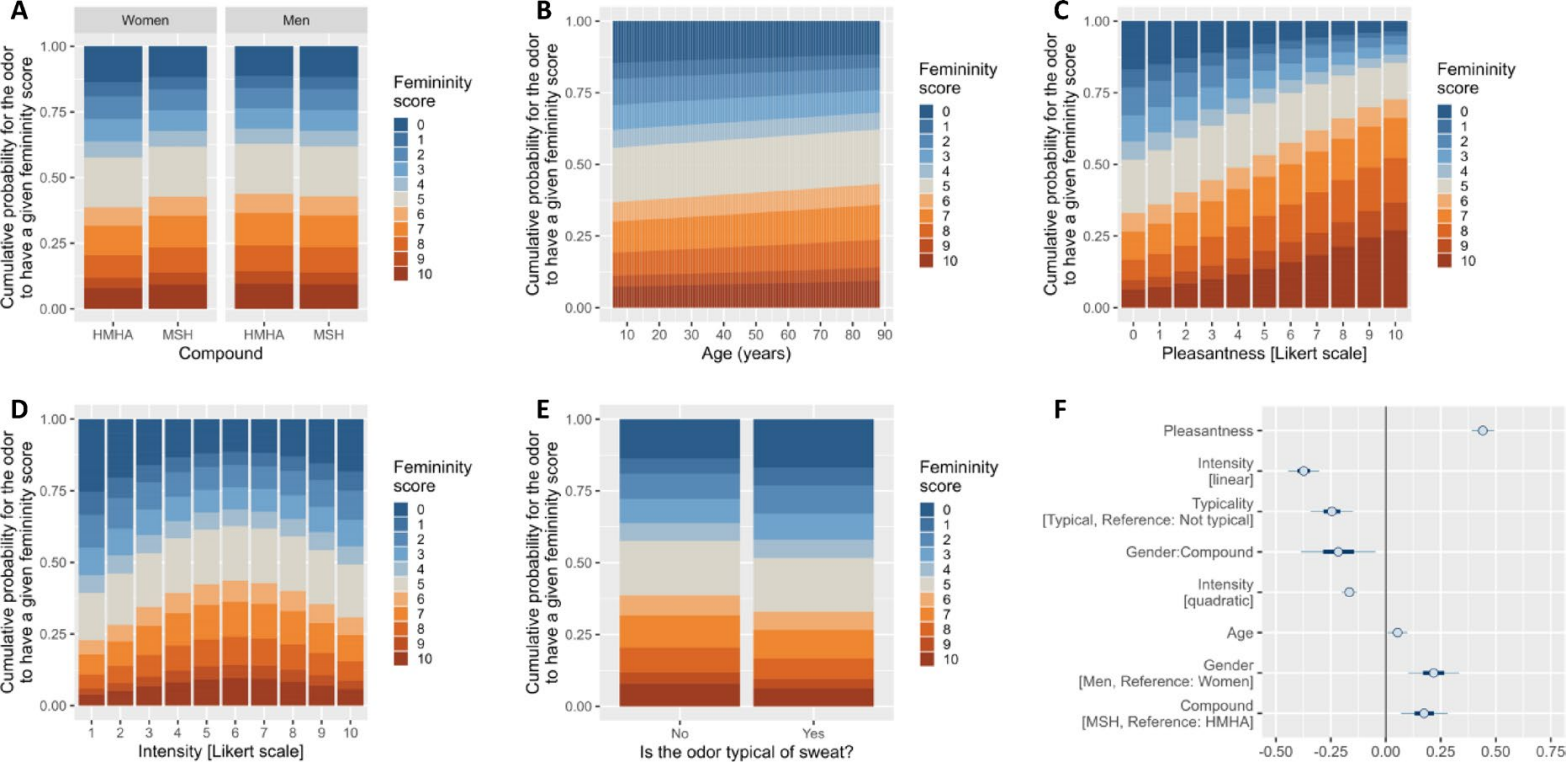
Effect of (**A**) the Rater Gender × Compound interaction, (**B**) Rater Age, (**C**) Pleasantness rating, (**D**) Intensity rating and (**E**) Typicality on Masculinity/Femininity ratings (0 = Masculine in blue, 10 = Feminine in orange; expressed in cumulative probability to receive a given score). (**F**) Log odds-ratio for each variable retained in the reduced model, with the Masculinity/Femininity rating of the odor as the response variable. Dots, thick lines and thin lines represent the mean, the 50% Credible Interval [CI] and the 90% CI, respectively. Thin lines not crossing the black vertical line at x = 0 indicate that at least 90% of the posterior distribution of that variable has the same sign (which is equivalent to a significance level of 0.10 in the frequentist framework). For continuous variables (e.g., Pleasantness), positive posterior distributions indicate that high values on that variable are associated with high ratings of femininity. Conversely, negative posterior distributions indicate that high values on that variable are associated with low ratings of femininity. For categorical variables (e.g., Gender), positive posterior distributions indicate that the category that is not the reference (e.g., Men) give higher ratings of femininity than the reference category. Conversely, negative posterior distributions indicate that the category that is not the reference give lower ratings of femininity than the reference category. Interactions and quadratic terms are easier to interpret by looking at panels A and D. Variables are sorted according to the absolute value of their Cohen’s *d* (highest on top).

For our main rating of interest, that is Masculinity/Femininity, we found a significant Rater Gender × Compound interaction (Est. = -0.22 ± 0.10, 90% CI [-0.38; -0.04], p_-_ = 0.9826; Table 2). Women were slightly more likely to rate MSH as more feminine (less masculine) than HMHA while there was no difference between the two compounds in male raters (Fig. 2A). There was also an effect of Rater Age (Est. = 0.05 ± 0.03, 90% CI [0.01; 0.10], p_+_ = 0.9756; Table 2). Older participants rated the odors as more feminine (less masculine) than younger ones (Fig. 2B). However, these effects remain very small (absolute value of Cohen’s *d*s < 0.12; Table 2).

**Fig. 3:**
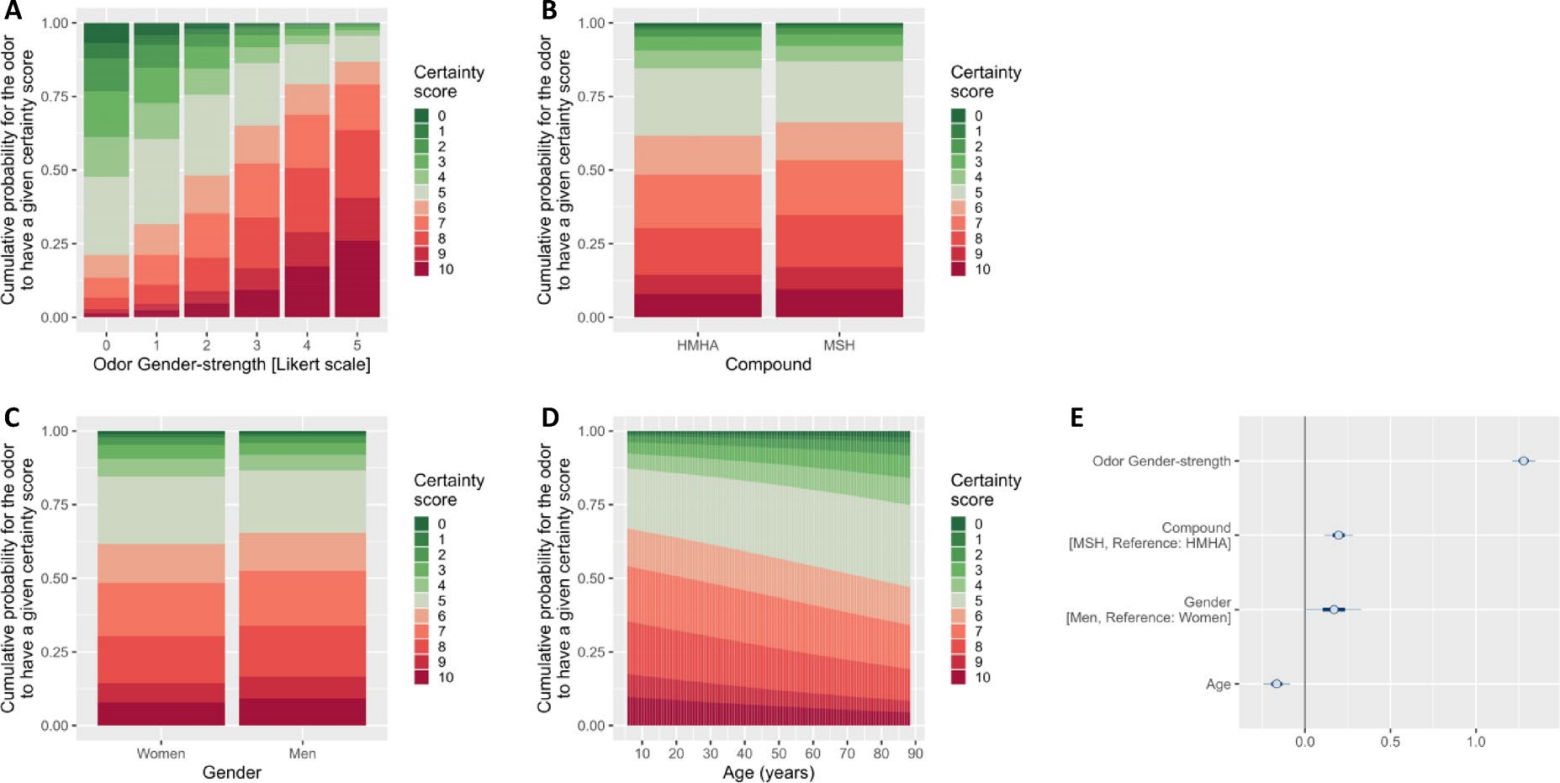
Effect of (**A**) Odor Gender-strength, (**B**) Compound, (**C**) Rater Gender, (**D**) Rater Age on the ratings of Certainty of the Masculinity/Femininity response (from 0 = low to 10 = high; expressed in cumulative probability to receive a given score). (**E**) Log odds-ratio for each variable retained in the reduced model, with the Certainty rating as the response variable. Dots, thick lines and thin lines represent the mean, the 50% Credible Interval [CI] and the 90% CI, respectively (see legend of Fig. 2 for more details). Variables are sorted according to the absolute value of their Cohen’s *d* (highest on top).

We further qualified these results with the rating of Certainty. The participants were more confident in their Masculinity/Femininity rating when they perceived the odor as very gendered (very masculine or very feminine) than when they rated it as gender-neutral (Odor Gender-strength: Est. = 1.28 ± 0.04, 90% CI [1.21; 1.35], p_+_ = 1; Table 2; Fig. 3A).This effect was of medium size (Cohen’s *d* = 0.71; Table 2). Confidence was higher for MSH than for HMHA (Fig. 3B; see Fig. S1 for illustration of compound differences in all variables). Also, confidence was higher for men than for women (Fig. 3C), and decreased with age (Fig. 3D). These effects remained, however, very small (Rater Gender effect: Est. = 0.17 ± 0.10, 90% CI [0.01; 0.33], p_+_ = 0.9575; Compound effect: Est. = 0.20 ± 0.05, 90% CI [0.11; 0.28], p_+_ = 1; Rater Age effect: Est. = -0.17 ± 0.05, 90% CI [-0.25; -0.09], p_-_ = 0.9996; Cohen’s *d*s < 0.11; Table 2).

As for the other odor ratings, we found that the most prominent predictor of Masculinity/Femininity rating was Pleasantness (Est. = 0.44 ± 0.03, 90% CI [0.39; 0.49], p_+_ = 1; Table 2), with a small effect size (Cohen’s *d* = 0.24; Table 2). The more pleasant the odor, the more likely it was to be evaluated as feminine (Fig. 2C). Some other effects were found, although they were very small (Cohen’s *d*s < 0.14). First, there was an inverted U-shape relationship between Intensity and Masculinity/Femininity ratings (Est. = -0.17 ± 0.02, 90% CI [-0.20; -0.13], p_-_ = 1; Table 2). Odors perceived as being of medium intensity (score around 5) were equally likely to be rated feminine or masculine, whereas odors perceived as being of low or high intensity were rated as being more masculine (Fig. 2D). Second, odors that were perceived as being more typical of sweat were rated as more masculine than odors perceived as being less typical of sweat (Est. = -0.24 ± 0.06, 90% CI [-0.34; -0.15], p_-_ = 0.9999; Table 2; Fig. 2E).

**Table 2:** Results of the reduced Bayesian linear mixed model on Masculinity/Femininity ratings and on certainty of the Masculinity/Femininity rating.

Variable	Estimate [median]	Estimate error [MAD]	90% CI [low]	90% CI [high]	p_+_ or p_-_	Cohen’s d [median]	Cohen’s d [MAD]
**Masculinity/Femininity**							
Pleasantness	0.44	0.03	0.39	0.49	1.0000	0.2433	0.0171
Intensity [linear]	-0.37	0.04	-0.44	-0.30	1.0000	-0.2058	0.0236
Typicality [ref: Not typical]	-0.24	0.06	-0.34	-0.15	0.9999	-0.1351	0.0317
Rater Gender × Compound	-0.22	0.10	-0.38	-0.04	0.9826	-0.1191	0.0575
Intensity [quadratic]	-0.17	0.02	-0.20	-0.13	1.0000	-0.0914	0.0115
Rater Age	0.05	0.03	0.01	0.10	0.9756	0.0296	0.0117
Rater Gender [ref: Women]	0.22	0.07	0.10	0.33	Int	Int	Int
Compound [ref: HMHA]	0.17	0.06	0.07	0.28	Int	Int	Int
Rater Age × Compound	–	–	–	–	–	–	–
Familiarity	–	–	–	–	–	–	–
Rater Age × Rater Gender × Compound	–	–	–	–	–	–	–
**Certainty of the Masculinity/Femininity rating**							
Odor Gender-strength	1.28	0.04	1.21	1.35	1.0000	0.7054	0.0232
Compound [ref: HMHA]	0.20	0.05	0.11	0.28	1.0000	0.1083	0.0281
Rater Gender [ref: Women]	0.17	0.10	0.01	0.33	0.9575	0.0936	0.0537
Rater Age	-0.17	0.05	-0.25	-0.09	0.9996	-0.0912	0.0266
Rater Gender × Compound	–	–	–	–	–	–	–
Rater Age × Compound	–	–	–	–	–	–	–
Odor Gender-strength × Compound	–	–	–	–	–	–	–
Rater Age × Rater Gender × Compound	–	–	–	–	–	–	–

The items in the “Variable” column are fixed factors, and SubjectID (with a random slope for Compound) and presentation order were used as random factors. MAD: Median Absolute Deviation, CI: Bayesian 90% Credible Interval (low and high boundaries), p_+_ or p_-_: probability that the posterior distribution has the same effect direction than the estimate. Variables are sorted according to the absolute value of their Cohen’s *d*. Main effects involved in interactions (Int) are presented but not interpreted. Higher values of the estimates indicate higher probabilities to rate an odor as more feminine. Lines with minus (–) signs display variables that were removed in the reduced model. For each variable, the estimate and the limits of the 90% CI correspond respectively to the dot and the limits of the thin line in Fig. 2F.

### Pleasantness, intensity, familiarity

**Fig. 4:**
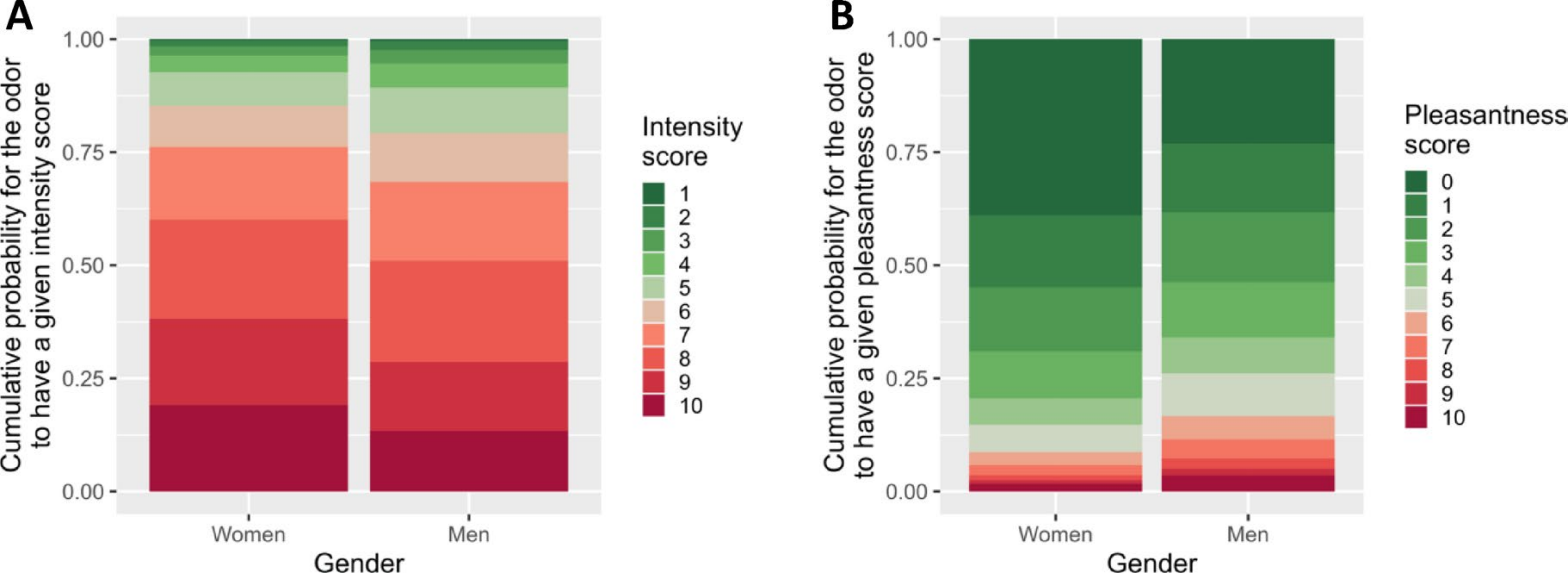
Effect of Rater Gender on Intensity (**A**) and Pleasantness ratings (**B**). Ratings (0 = low, 10 = high for Pleasantness; 1 = low, 10 = high for Intensity) are expressed in cumulative probability to receive a given score.

In this section focusing on the other odor ratings, only effects with Cohen’s *d* > 0.2 are illustrated (Fig. 4; see Fig. S2 for the smaller effects). For Intensity and Pleasantness, the interactions Rater Gender × Compound and Rater Age × Rater Gender × Compound were not significant. There were only main effects of Gender for Intensity (Est. = -0.42 ± 0.09, 90% CI [-0.56; -0.28], p_-_ = 1; Table 3) and – with the biggest effect size (Cohen’s *d* = 0.40) – for Pleasantness (Est. = 0.73 ± 0.10, 90% CI [0.57; 0.89], p_+_ = 1; Table 3). More specifically, women provided higher intensity (Fig. 4A) and lower pleasantness ratings than men (Fig. 4B), independently of the odor compound sampled. We found a significant Rater Age × Compound interactions for Intensity (Est. = 0.26 ± 0.06, 90% CI [0.17; 0.36], p_+_ = 1; Table 3), and for Pleasantness (Est. = -0.31 ± 0.06, 90% CI [-0.41; -0.21], p_-_ = 1; Table 3). The perceived intensity tended to increase with age for MSH and to decrease for HMHA (Fig. S2A). Pleasantness tended to decrease with age for MSH while the reverse occurred for HMHA (Fig. S2B).

For familiarity, the 3-way interaction Rater Age × Rater Gender × Compound was significant, with a very small effect size (Est. = 0.25 ± 0.12, 90% CI [0.05; 0.44], p_+_ = 0.98, Cohen’s *d* = 0.14; Table 3). The perceived familiarity of both compounds was lower for men than for women and increased with age, and MSH elicited higher familiarity ratings than HMHA (Fig. S2C). The triple interaction seems to be due to the fact that, compared to the three other cases, the increase of familiarity with age was sharpest for men rating HMHA (Fig. S2C).

**Table 3:** Results of the three reduced Bayesian linear mixed models on Intensity, Familiarity, and Pleasantness.

Variable	Estimate [median]	Estimate error [MAD]	90% CI [low]	90% CI [high]	*p*_+_ or *p*_-_	Cohen’s d [median]	Cohen’s d [MAD]
**Intensity**							
Rater Gender [ref: Women]	-0.42	0.09	-0.56	-0.28	1.0000	-0.2310	0.0476
Rater Age × Compound	0.26	0.06	0.17	0.36	1.0000	0.1445	0.0310
Rater Age	-0.05	0.05	-0.13	0.04	Int	Int	Int
Compound [ref: HMHA]	0.49	0.06	0.40	0.60	Int	Int	Int
Rater Gender × Compound	–	–	–	–	–	–	–
Rater Age × Rater Gender × Compound	–	–	–	–	–	–	–
**Familiarity**							
Rater Age × Gender × Compound	0.25	0.12	0.05	0.44	1.0000	0.1356	0.0652
Compound [ref: HMHA]	0.49	0.07	0.38	0.59	Int	Int	Int
Rater Gender [Ref: Women]	-0.36	0.12	-0.55	-0.17	Int	Int	Int
Rater Age	0.32	0.07	0.20	0.44	Int	Int	Int
Rater Age × Compound	-0.05	0.07	-0.16	0.05	Int	Int	Int
Rater Gender × Compound	0.08	0.10	-0.09	0.25	Int	Int	Int
**Pleasantness**							
Rater Gender [ref: Women]	0.73	0.10	0.57	0.89	1.0000	0.4031	0.0531
Rater Age × Compound	-0.31	0.06	-0.41	-0.21	1.0000	-0.1706	0.0330
Rater Age	0.22	0.05	0.13	0.30	Int	Int	Int
Compound [ref: HMHA]	-0.20	0.08	-0.33	-0.07	Int	Int	Int
Rater Gender × Compound	–	–	–	–	–	–	–
Rater Age × Rater Gender × Compound	–	–	–	–	–	–	–

The items in the “Variable” column are fixed factors, and SubjectID (with a random slope for Compound) and presentation order were used as random factors. MAD: Median Absolute Deviation, CI: Bayesian 90% Credible Interval (low and high boundaries), p_+_ or p_-_: probability that the posterior distribution has the same effect direction than the estimate. Variables are sorted according to the absolute value of their Cohen’s *d*. Main effects involved in interactions (Int) are presented but not interpreted. Higher values of the estimates indicate higher probabilities to rate an odor as more intense, familiar, pleasant, or to rate the odor femininity/masculinity with higher certainty, respectively. Lines with minus (–) signs display variables that were removed in the reduced model. For each variable, the estimate and the limits of the 90% CI correspond respectively to the dot and the limits of the thin line in Fig. S3.

## Discussion

This study aimed to investigate how two body odor compounds, reported to be emitted in sexually dimorphic amounts (the “feminine” compound MSH and the “masculine” compound HMHA), are perceived. To this end, we had these odors evaluated by a very large sample of men and women of all ages attending a museum exhibition.

The main question tested was whether MSH was perceived as more feminine than HMHA, and conversely HMHA as more masculine than MSH. We found this to be the case only in women, although they were less confident in their ratings than men. In two other studies, different outcomes were found. However, they used much smaller sample sizes (*N* = 20 women, 19 men^27^, and *N* = 15 women, 15 men^29^) and different contexts of odor presentation (implicit during a face rating task^27^ or totally decontextualized^29^). In one study, men (but not women) perceived HMHA as more masculine than women did, while no difference was found for MSH^29^. In the other using only HMHA, no gender differences were found for Masculinity/Femininity ratings^27^. This suggests that the explicit evaluation of the masculine or feminine character of these odors is easily fluctuating, surely depending on the context.

The result of the present study needs to be further qualified, for two reasons. First, the effect size is very small despite the very large sample size (*N* = 2,716). This is due to a very high interindividual variability in the Masculinity/Femininity ratings. Second, the scores are fairly non-gendered for both compounds (i.e., mean score around 5) (see Supplementary Table S1). If we assume that this effect is of practical interest, it seems likely that it reflects a better ability of women to process body odors, and odors in general. Previous studies have shown that women generally outperform men in all olfactory abilities^30^. In particular, they are better at performing fine discrimination of body odors (e.g., recognizing the odor of familiar individuals^17,38)^. This interpretation of our results is supported by several additional observations in our study. Firstly, we found that women rated the odors (without distinction between the compounds) as more intense, more familiar and less pleasant than the men. This is consistent with previous recurrent findings on body odors^16,43,52^ and unpleasant non-body odors^37^. Secondly, other than for Masculinity/Femininity, no interaction was found between gender and compound in this study, casting doubt on the possible role of HMHA and/or MSH compounds in mate preferences. In particular, one could have expected that MSH would be more pleasant to men and/or that HMHA would be more pleasant to women, which was not observed here. This result is in line with our previous studies, conducted on much smaller samples and in a non-contextualized manner (unlike here). In these studies, HMHA was found to be less^27^ or equally pleasant and attractive^28,29^ to women than to men. It also had no gender-specific effects on the perception of faces in more implicit approaches^27^. No gender differences were found either for MSH perception^29^.

Finally, the small effect size and huge variability observed in gender ratings of MSH and HMHA in our study may question our choice of these particular compounds. The quantities of these compounds’ precursors were described as highly variable from one individual to another, and as largely overlapping between men’s and women’s sample^24^. This may explain why the perceptual outcome was not obvious here. Clearer effects might have been found using more realistic mixtures of HMHA + MSH instead of single compounds presented separately. Indeed, male and female ratios were reported to be stable and 3 times higher in men^24^. Also, let us bear in mind that HMHA and MSH were not found as the most sexually dimorphic molecules *among* a series of body odor compounds. Very few other studies used this strategy, e.g.,^21^ who reported a series of 15 ‘gender markers’ found in complex body odor profiles. Rather, sex differences happened to have been found for those compounds during their study as malodors, what is more in a limited number of participants (24 men, 25 women). Consequently, it may be that other compounds (possibly not identified yet) may be more sexually dimorphic. These would be more relevant and might trigger more differentiated gender ratings. A promising future path to identify such compounds could be to use chemical analysis to identify the overall changes in body odor composition between pre- and post-puberty^53^. Other compounds of interest would be those for which sexual dimorphism increases after puberty.

As to the developmental effects of odor perception, we found that, with increasing age, the odors were rated as more feminine (although with lower levels of confidence) and more familiar, probably due to increasing exposure^54^. Also, although difficult to interpret, a different pattern of perceptual changes throughout life was found for MSH. It became more intense and more unpleasant with age. On the contrary, HMHA became less intense and less unpleasant with age. The latter effect is consistent with the presbyosmia and reduced negativity bias phenomena in aging^55,56^. Age related effects in our study may also result from shifts occurring earlier in development (e.g., during adolescence), as reported by several studies on body odors^57^ and body odor compounds^58^. Importantly, however, no interaction between age and compound was found for the variable Masculinity/Femininity. This again does not allow us to argue in favor of a function of these compounds in mate preferences.

Collecting our data in a museum during an exhibition was a considerable asset in terms of sample size and of representativeness of all age groups. It may also help diluting the effects of factors that we have chosen not to control, for parsimony reasons (non-binary gender identity, sexual orientation, etc.). Such a methodological choice also has its drawbacks, especially regarding the relative lack of control compared to a laboratory setting. We limited as much as possible the bias related to this lack of control. First, a priori, by placing the odors in hermetically sealed jars that were carefully watched over by the museum staff who refreshed the odors every two days. Second, *a posteriori*, by removing the obviously irrelevant responses (see excluded data in Participants Sect.). It was not possible to control for all sources of noise in the data though, such as the possible influence of people around the person being tested. However, the following findings are highly consistent with the literature, which suggests that we can trust the quality of the collected data. (i) The developmental course of HMHA and MSH perception described in the previous paragraph (Fig. S2B, C) is consistent with the well-known positive relationship between intensity and unpleasantness of body and non-body odors^59–61^. (ii) The significant increase of familiarity of the odors with age (Fig. S2C) is highly consistent with a well-documented effect of exposure^54^. (iii) The prominent gender differences found for several perceptual variables are similar to those consistently reported in the literature on olfaction (see^30^ for a review).

To summarize, women perceived HMHA as more masculine than MSH in accordance with the alleged sexual dimorphism of these compounds. However, this effect was very small and there was little consensus in the cohort (huge variability) on the gender rating of these compounds. The results agree with the existing literature regarding women’s advantage in (social) odor processing and age-related changes in perceptual responses, but fail to support the hypothesis that HMHA and/or MSH could be involved in mate preferences. Several research perspectives can be proposed to better understand the olfactory determinants of male-female attractiveness^20^. For example, a better knowledge of the chemical composition of human body odor (and of its variations as a function of gender, hormonal status and age) would help identifying other possible relevant compounds. Also, we recommend using more ecological approaches where compounds are presented as mixtures (e.g., HMHA/MSH in a certain ratio) or embedded in a “baseline body odor”, rather than in an isolated manner. Finally, experimental designs testing implicit effects of odors, on behavior or physiology for instance, may provide valuable information.

## Supplementary Information

Below is the link to the electronic supplementary material.

Supplementary Material 1

## Data Availability

Data and analyses scripts can be downloaded at https://osf.io/hdtm7/.

## References

[1] J. Havlíček, J. Fialová, S.C. Roberts, Individual variation in body odor, in: A. Buettner (Ed.), Springer Handb. Odor, Springer International Publishing, Cham, pp. 125–126. 10.1007/978-3-319-26932-0_50. (2017).

[2] C. Wedekind, T. Seebeck, F. Bettens, A.J. Paepke, MHC-dependent mate preferences in humans, *Proc. R. Soc. Lond. B Biol. Sci.* 260 245–249. 10.1098/rspb.1995.0087. (1995). 10.1098/rspb.1995.0087 7630893

[3] J. Winternitz, J.L. Abbate, E. Huchard, J. Havlíček, L.Z. Garamszegi, Patterns of MHC-dependent mate selection in humans and nonhuman primates: a meta-analysis, *Mol. Ecol.* 26 668–688. 10.1111/mec.13920. (2017). 27859823 10.1111/mec.13920

[4] D. Singh, P.M. Bronstad, Female body odour is a potential cue to ovulation, *Proc. R. Soc. Lond. B Biol. Sci.* 268, 797–801. 10.1098/rspb.2001.1589. (2001). 10.1098/rspb.2001.1589 PMC1088671 11345323

[5] S. Kuukasjarvi, C.J.P. Eriksson, E. Koskela, T. Mappes, K. Nissinen, M.J. Rantala, Attractiveness of women’s body odors over the menstrual cycle: the role of oral contraceptives and receiver sex, *Behav. Ecol.* 15, 579–584. 10.1093/beheco/arh050. (2004).

[6] M.J. Rantala, C.J.P. Eriksson, A. Vainikka, R. Kortet, Male steroid hormones and female preference for male body odor, *Evol. Hum. Behav.* 27, 259–269. 10.1016/j.evolhumbehav.2005.11.002. (2006).

[7] M.J. Olsson, J.N. Lundström, B.A. Kimball, A.R. Gordon, B. Karshikoff, N. Hosseini, K. Sorjonen, C.O. Höglund, C. Solares, A. Soop, J. Axelsson, M. Lekander, The scent of disease: human body odor contains an early chemosensory cue of sickness, *Psychol. Sci.* 25, 817–823. 10.1177/0956797613515681. (2014). 24452606 10.1177/0956797613515681

[8] A. Sorokowska, P. Sorokowski, A. Szmajke, Does personality smell? Accuracy of personality assessments based on body odour, *Eur. J. Personal.* 26, 496–503. 10.1002/per.848. (2012).

[9] J.H.B. de Groot, M.A.M. Smeets, Human fear chemosignaling: evidence from a meta-analysis, *Chem. Senses* 42, 663–673. 10.1093/chemse/bjx049. (2017). 28981822 10.1093/chemse/bjx049

[10] S. Richard Ortegón, A. Fournel, O. Carlos, K. Kawabata Duncan, K. Hirabayashi, K. Tagai, A. Abriat, M. Bensafi, B. Race, C. Ferdenzi, And I’m feeling good: effect of emotional sweat and perfume on others’ physiology, verbal responses, and creativity, *Chem. Senses* 47, bjac012. 10.1093/chemse/bjac012. (2022). 35588293 10.1093/chemse/bjac012

[11] I. Ravreby, K. Snitz, N. Sobel, There is chemistry in social chemistry, *Sci. Adv.* 8, eabn0154. 10.1126/sciadv.abn0154.(2022). 35749498 10.1126/sciadv.abn0154 PMC9232116

[12] M. Gallagher, C.J. Wysocki, J.J. Leyden, A.I. Spielman, X. Sun, G. Preti, Analyses of volatile organic compounds from human skin, *Br. J. Dermatol.* 159, 780–791. 10.1111/j.1365-2133.2008.08748.x. (2008). 18637798 10.1111/j.1365-2133.2008.08748.x PMC2574753

[13] S. Haze, Y. Gozu, S. Nakamura, Y. Kohno, K. Sawano, H. Ohta, K. Yamazaki, 2-Nonenal newly found in human body odor tends to increase with aging, *J. Invest. Dermatol.* 116, 520–524. 10.1046/j.0022-202x.2001.01287.x. (2001). 11286617 10.1046/j.0022-202x.2001.01287.x

[14] D. Chen, J. Haviland-Jones, Rapid mood change and human odors, *Physiol. Behav.* 68, 241–250. (1999). 10627087 10.1016/s0031-9384(99)00147-x

[15] S. Mitro, A.R. Gordon, M.J. Olsson, J.N. Lundström, The smell of age: perception and discrimination of body odors of different ages, *PLoS One* 7, e38110. 10.1371/journal.pone.0038110. (2012). 22666457 10.1371/journal.pone.0038110 PMC3364187

[16] R.L. Doty, P.A. Green, C. Ram, S.L. Yankell, Communication of gender from human breath odors: relationship to perceived intensity and pleasantness, *Horm. Behav.* 16, 13–22. 10.1016/0018-506X(82)90002-2. (1982). 7068124 10.1016/0018-506x(82)90002-2

[17] M. Schleidt, B. Hold, G. Attili, A cross-cultural study on the attitude towards personal odors, *J. Chem. Ecol.* 7, 19–31. 10.1007/BF00988632. (1981). 24420424 10.1007/BF00988632

[18] R.L. Doty, M.M. Orndorff, J. Leyden, A. Kligman, Communication of gender from human axillary odors: relationship to perceived intensity and hedonicity, *Behav. Biol.* 23, 373–380. (1978). 697690 10.1016/s0091-6773(78)91393-7

[19] L. Dormont, J.-M. Bessière, A. Cohuet, Human skin volatiles: a review, *J. Chem. Ecol.* 39, 569–578. 10.1007/s10886-013-0286-z. (2013). 23615881 10.1007/s10886-013-0286-z

[20] C. Ferdenzi, S. Richard Ortegón, S. Delplanque, N. Baldovini, M. Bensafi, Interdisciplinary challenges for elucidating human olfactory attractiveness, *Philos. Trans. R. Soc. B Biol. Sci.* 375 20190268. 10.1098/rstb.2019.0268. (2020). 10.1098/rstb.2019.0268 PMC7209927 32306873

[21] D.J. Penn, E. Oberzaucher, K. Grammer, G. Fischer, H.A. Soini, D. Wiesler, M.V. Novotny, S.J. Dixon, Y. Xu, R.G. Brereton, Individual and gender fingerprints in human body odour, *J. R. Soc. Interface* 4, 331–340. 10.1098/rsif.2006.0182. (2007). 17251141 10.1098/rsif.2006.0182 PMC2359862

[22] S. Bird, D.B. Gower, The validation and use of a radioimmunoassay for 5 alpha-androst-16-en-3-one in human axillary collections, *J. Steroid Biochem.* 14, 213–219. 10.1016/0022-4731(81)90176-X. (1981). 7193782 10.1016/0022-4731(81)90176-x

[23] V. Brémond Bostoen, S. Richard Ortegón, N. Barthès, B. Buatois, F. Nicolè, D. Steyer, L. Dormont, C. Ferdenzi, ABOV: A novel system of direct headspace skin sampling to study human body odor, *J. Chem. Ecol.* 51, 31. 10.1007/s10886-025-01581-7. (2025). 40056297 10.1007/s10886-025-01581-7

[24] M. Troccaz, G. Borchard, C. Vuilleumier, S. Raviot-Derrien, Y. Niclass, S. Beccucci, C. Starkenmann, Gender-specific differences between the concentrations of nonvolatile (R)/(S)-3-methyl-3-sulfanylhexan-1-ol and (R)/(S)-3-hydroxy-3-methyl-hexanoic acid odor precursors in axillary secretions, *Chem. Senses* 34, 203–210. 10.1093/chemse/bjn076. (2009). 19147808 10.1093/chemse/bjn076

[25] P. Jackman, W. Noble, Normal axillary skin microflora in various populations, *Clin. Exp. Dermatol.* 8, 259–268. 10.1111/j.1365-2230.1983.tb01778.x. (1983). 6411399 10.1111/j.1365-2230.1983.tb01778.x

[26] A. Natsch, H. Gfeller, P. Gygax, J. Schmid, G. Acuna, A specific bacterial aminoacylase cleaves odorant precursors secreted in the human axilla,* J. Biol. Chem.* 278, 5718–5727. 10.1074/jbc.M210142200. (2003). 12468539 10.1074/jbc.M210142200

[27] C. Ferdenzi, A. Fournel, N. Baldovini, D. Poupon, D. Ligout, M. Thévenet, R. Bouet, M. Bensafi, Influence of the human body odor compound HMHA on face perception, *Perception *10.1177/03010066231222473. (2024). 10.1177/03010066231222473 38216326

[28] C. Ferdenzi, H. Razafindrazaka, N. Baldovini, D. Poupon, D. Pierron, M. Bensafi, Influence of gender and culture on the perception of acidic compounds of human body odor, *Physiol. Behav.* 210, 112561. 10.1016/j.physbeh.2019.112561. (2019). 31178171 10.1016/j.physbeh.2019.112561

[29] C. Ferdenzi, A. Fournel, L. Fantin, S.R. Ortegón, C. Manesse, N. Baldovini, M. Thévenet, F. Lamberton, D. Ibarrola, F. Faure, M. Bensafi, Neural representation of allegedly sex-specific human body odor compounds, *NeuroImage* 310121114. 10.1016/j.neuroimage.2025.121114. (2025). 40086707 10.1016/j.neuroimage.2025.121114

[30] P. Sorokowski, M. Karwowski, M. Misiak, M.K. Marczak, M. Dziekan, T. Hummel, A. Sorokowska, Sex differences in human olfaction: A Meta-Analysis, *Front. Psychol.* 10, 242. 10.3389/fpsyg.2019.00242. (2019). 30814965 10.3389/fpsyg.2019.00242 PMC6381007

[31] M. Larsson, M. Lovden, L.-G. Nilsson, Sex differences in recollective experience for olfactory and verbal information, *Acta Psychol. (Amst.)* 112, 89–103. (2003). 12423901 10.1016/s0001-6918(02)00092-6

[32] R.L. Doty, P. Shaman, S.L. Applebaum, R. Giberson, L. Siksorski, L. Rosenberg, Smell identification ability: changes with age, *Science* 226, 1441–1443. 10.1126/science.6505700. (1984). 6505700 10.1126/science.6505700

[33] S. Nordin, M. Bende, E. Millqvist, Normative data for the chemical sensitivity scale, *J. Env. Psychol*. 24, 399–403. (2004).

[34] A. Wrzesniewski, C. McCauley, P. Rozin, Odor and affect: individual differences in the impact of odor on liking for places, things and people, *Chem. Senses* 24, 713–721. (1999). 10587506 10.1093/chemse/24.6.713

[35] J. Havlicek, T.K. Saxton, S.C. Roberts, E. Jozifkova, S. Lhota, J. Valentova, J. Flegr, He sees, she smells? Male and female reports of sensory reliance in mate choice and non-mate choice contexts, *Personal. Individ. Differ.* 45, 565–570. 10.1016/j.paid.2008.06.019. (2008).

[36] R.S. Herz, M. Inzlicht, Sex differences in response to physical and social factors involved in human mate selection: the importance of smell for women, *Evol. Hum. Behav.* 23, 359–364. 10.1016/s1090-5138(02)00095-8. (2002).

[37] J.K. Olofsson, S. Nordin, Gender differences in chemosensory perception and event-related potentials, *Chem. Senses* 29, 629–637. (2004). 15337687 10.1093/chemse/bjh066

[38] C. Ferdenzi, B. Schaal, S.C. Roberts, Family scents: developmental changes in the perception of kin body odor?, *J. Chem. Ecol.* 36, 847–854. 10.1007/s10886-010-9827-x. (2010). 20640943 10.1007/s10886-010-9827-x

[39] S.A. Collins, Men’s voices and women’s choices, *Anim. Behav.* 60, 773–780. 10.1006/anbe.2000.1523. (2000). 11124875 10.1006/anbe.2000.1523

[40] A.C. Little, T.K. Saxton, S.C. Roberts, B.C. Jones, L.M. Debruine, J. Vukovic, D.I. Perrett, D.R. Feinberg, T. Chenore, Women’s preferences for masculinity in male faces are highest during reproductive age range and lower around puberty and post-menopause, *Psychoneuroendocrinology* 35, 912–920. 10.1016/j.psyneuen.2009.12.006. (2010). 20060226 10.1016/j.psyneuen.2009.12.006

[41] C. Ferdenzi, S. Delplanque, R. Atanassova, D. Sander, Androstadienone’s influence on the perception of facial and vocal attractiveness is not sex specific, *Psychoneuroendocrinology* 66, 166–175. 10.1016/j.psyneuen.2016.01.016. (2016). 26827295 10.1016/j.psyneuen.2016.01.016

[42] T.D. Wyatt, The search for human pheromones: the lost decades and the necessity of returning to first principles, *Proc. R. Soc. Lond. B Biol. Sci.* 282, 20142994. 10.1098/rspb.2014.2994. (2015). 10.1098/rspb.2014.2994 PMC4375873 25740891

[43] R.J. Stevenson, B.M. Repacholi, Age-related changes in children’s hedonic response to male body odor, *Dev. Psychol.* 39, 670–679. (2003). 12859121 10.1037/0012-1649.39.4.670

[44] T. Hummel, B. Sekinger, S.R. Wolf, E. Pauli, G. Kobal, “Sniffin’ sticks”: olfactory performance assessed by the combined testing of odor identification, odor discrimination and olfactory threshold, *Chem. Senses* 22, 39–52. 10.1093/chemse/22.1.39. (1997). 9056084 10.1093/chemse/22.1.39

[45] R core team, R: a language and environment for statistical computing, https://www.R-project.org/. (2021).

[46] P.-C. Bürkner, brms: an R package for Bayesian multilevel models using Stan, *J. Stat. Softw.* 80, 1–28. 10.18637/jss.v080.i01. (2017).

[47] A. Vehtari, A. Gelman, J. Gabry, Practical Bayesian model evaluation using leave-one-out cross-validation and WAIC, *Stat. Comput.* 27, 1413–1432. 10.1007/s11222-016-9696-4. (2017).

[48] J.S. Martin, S.E. Koski, T. Bugnyar, A.V. Jaeggi, J.J.M. Massen, Prosociality, social tolerance and partner choice facilitate mutually beneficial cooperation in common marmosets, *Callithrix jacchus*, *Anim. Behav.* 173, 115–136. 10.1016/j.anbehav.2020.12.016. (2021).

[49] D. Makowski, M.S. Ben-Shachar, S.H.A. Chen, D. Lüdecke, Indices of effect existence and significance in the Bayesian framework, *Front. Psychol.* 10, 2767. 10.3389/fpsyg.2019.02767. (2019). 31920819 10.3389/fpsyg.2019.02767 PMC6914840

[50] J. Cohen, Statistical Power Analysis for the Behavioral Sciences, 2nd ed., Academic Press, New York, (1988).

[51] J.A. Rosenthal, Qualitative descriptors of strength of association and effect size, *J. Soc. Serv. Res.* 21, 37–59. 10.1300/J079v21n04_02. (1996).

[52] C. Ferdenzi, S.C. Roberts, A. Schirmer, S. Delplanque, S. Cekic, C. Porcherot, I. Cayeux, D. Sander, D. Grandjean, Variability of affective responses to odors: Culture, gender, and olfactory knowledge, *Chem. Senses* 38, 175–186. 10.1093/chemse/bjs083. (2013). 23196070 10.1093/chemse/bjs083

[53] D. Owsienko, L. Goppelt, K. Hierl, L. Schäfer, I. Croy, H.M. Loos, Body odor samples from infants and post-pubertal children differ in their volatile profiles, *Commun. Chem.* 7, 1–10. 10.1038/s42004-024-01131-4. (2024). 38514840 10.1038/s42004-024-01131-4 PMC10957943

[54] S. Delplanque, G. Coppin, L. Bloesch, I. Cayeux, D. Sander, The mere exposure effect depends on an odor’s initial pleasantness, *Front. Psychol.* 6, 911. 10.3389/fpsyg.2015.00920. (2015). 26191021 10.3389/fpsyg.2015.00920 PMC4490210

[55] R.L. Doty, V. Kamath, The influences of age on olfaction: a review, *Front. Psychol.* 5, 20. 10.3389/fpsyg.2014.00020. (2014). 24570664 10.3389/fpsyg.2014.00020 PMC3916729

[56] S. Vieillard, L. Ronat, A. Baccarani, B. Schaal, J.-Y. Baudouin, R. Brochard, Age differences in olfactory affective responses: evidence for a positivity effect and an emotional dedifferentiation, *Neuropsychol. Dev. Cogn. B Aging Neuropsychol. Cogn.* 28, 570–583. 10.1080/13825585.2020.1799926. (2021). 32787505 10.1080/13825585.2020.1799926

[57] L.M. Novakova, D. Plotena, J. Havlicek, Age and pubertal Status-Related changes in reports of perception of personal odors, *Perception* 46, 484–497. 10.1177/0301006616686096. (2017). 28056656 10.1177/0301006616686096

[58] T. Hummel, F. Krone, J.N. Lundström, O. Bartsch, Androstadienone odor thresholds in adolescents, *Horm Behav.* 47, 306–10. (2005). 15708759 10.1016/j.yhbeh.2004.10.007

[59] R.L. Doty, An examination of relationships between the pleasantness, intensity, and concentration of 10 odorous stimuli, *Percept. Psychophys.* 17, 492–496. 10.3758/BF03203300. 1975).

[60] H. Distel, S. Ayabe-Kanamura, M. Martinez-Gomez, I. Schicker, T. Kobayakawa, S. Saito, R. Hudson, Perception of everyday odors–correlation between intensity, familiarity and strength of hedonic judgement, *Chem. Senses* 24, 191–199. 10.1093/chemse/24.2.191. (1999). 10321820 10.1093/chemse/24.2.191

[61] J. Havlicek, P. Lenochova, The effect of meat consumption on body odor attractiveness, *Chem. Senses* 31, 747–752. 10.1093/chemse/bjl017. (2006). 16891352 10.1093/chemse/bjl017

