# Supplementary material for: The perception of gender in two allegedly sex-specific body odor compounds MSH and HMHA
Source: Sci Rep. 2025 Nov 27;15:42447. doi: 10.1038/s41598-025-26457-4 (PMC12661034; doi:10.1038/s41598-025-26457-4)
Supplement: Supplementary file 1 — Supplementary Material 1 [file 41598_2025_26457_MOESM1_ESM.docx]

**Supplementary Material**

**Figure S1.** Overview of the ratings of Masculinity/Femininity **(A)**, Certainty **(B)**, Pleasantness **(C)**, Intensity **(D)** and Familiarity **(E)** of HMHA and MSH separately. Ratings (1 = low, 10 = high for Intensity; 0 = low, 10 = high for the other ratings) are expressed in cumulative probability to receive a given score.

**Figure S2.** Effect of the Rater Age × Compound interaction on Intensity **(A)** and Pleasantness ratings **(B).** Effect of the Rater Age × Rater Gender × Compound interaction on Familiarity ratings **(C).** Ratings (0 = low, 10 = high for Familiarity and Pleasantness; 1 = low, 10 = high for Intensity) are expressed in cumulative probability to receive a given score.

**Figure S3.** Log odds-ratio for each variable retained in the reduced model, with the Intensity **(A)**, Familiarity **(B)** and Pleasantness of the odor **(C)** as the response variable. Dots, thick lines and thin lines represent the mean, the 50% Credible Interval [CI] and the 90% CI, respectively. Thin lines not crossing the black vertical line at x = 0 indicate that at least 90% of the posterior distribution of that variable has the same sign (which is equivalent to a significance level of 0.10 in the frequentist framework). Positive posterior distributions indicate that high values on that variable are associated with high ratings. Conversely, negative posterior distributions indicate that high values on that variable are associated with low ratings. Variables are sorted according to the absolute value of their Cohen’s d (highest on top).

**Table S1.** Mean ± Standard Deviation of the Masculinity/Femininity ratings and of the Certainty of the Masculinity/Femininity ratings for each compound (MSH, HMHA), by Rater Gender and Rater Age group and for All groups combined.

|  | Masculinity/Femininity | | Certainty of the Masculinity/Femininity rating | |
| --- | --- | --- | --- | --- |
|  | MSH | HMHA | MSH | HMHA |
| *Women* | *4.34 ± 3.38* | *4.17 ± 3.15* | *6.38 ± 3.11* | *5.98 ± 3.21* |
| 6 to 9 | 4.40 ± 3.83 | 3.87 ± 3.67 | 7.29 ± 3.17 | 6.92 ± 3.32 |
| 10 to 19 | 4.30 ± 3.27 | 4.00 ± 3.08 | 6.19 ± 3.09 | 5.93 ± 3.18 |
| 20 to 29 | 4.35 ± 3.25 | 4.15 ± 2.99 | 6.38 ± 3.12 | 5.92 ± 3.19 |
| 30 to 39 | 4.26 ± 3.36 | 4.22 ± 3.11 | 6.14 ± 3.05 | 5.66 ± 3.09 |
| 40 to 49 | 4.45 ± 3.38 | 4.15 ± 3.13 | 6.48 ± 3.11 | 6.33 ± 3.21 |
| 50 to 59 | 4.70 ± 3.72 | 4.53 ± 3.34 | 6.60 ± 3.19 | 5.70 ± 3.24 |
| 60 to 69 | 4.20 ± 3.44 | 4.99 ± 3.33 | 6.84 ± 2.83 | 5.93 ± 3.25 |
| 70 to 90 | 3.62 ± 2.59 | 4.23 ± 2.55 | 4.77 ± 3.39 | 5.31 ± 3.64 |
| *Men* | *4.55 ± 3.36* | *4.75 ± 3.15* | *6.56 ± 2.97* | *6.20 ± 3.03* |
| 6 to 9 | 4.59 ± 4.04 | 4.99 ± 3.74 | 7.41 ± 3.21 | 6.81 ± 3.46 |
| 10 to 19 | 4.36 ± 3.30 | 4.44 ± 3.26 | 6.82 ± 2.91 | 6.69 ± 2.96 |
| 20 to 29 | 4.81 ± 3.31 | 5.17 ± 2.98 | 6.47 ± 2.87 | 6.06 ± 2.83 |
| 30 to 39 | 4.70 ± 3.20 | 4.85 ± 2.86 | 5.95 ± 2.96 | 5.61 ± 2.92 |
| 40 to 49 | 4.15 ± 3.15 | 4.55 ± 3.01 | 6.49 ± 2.83 | 6.05 ± 2.97 |
| 50 to 59 | 5.55 ± 3.42 | 4.74 ± 3.17 | 6.49 ± 3.06 | 6.35 ± 3.03 |
| 60 to 69 | 3.97 ± 3.31 | 4.79 ± 2.91 | 6.38 ± 2.87 | 5.24 ± 2.95 |
| 70 to 90 | 3.53 ± 3.06 | 5.00 ± 3.30 | 5.79 ± 3.28 | 5.05 ± 3.47 |
| *All* | *4.42 ± 3.37* | *4.39 ± 3.16* | *6.44 ± 3.06* | *6.07 ± 3.14* |

**Table S2.** Mean ± Standard Deviation of the Intensity, Familiarity and Pleasantness ratings, and % “yes” responses to the question “does this correspond to a typical smell of sweat?” for each compound (MSH, HMHA), by Rater Gender and Rater Age group and for All groups combined.

|  | Intensity | | Familiarity | | Pleasantness | | Typical of sweat | |
| --- | --- | --- | --- | --- | --- | --- | --- | --- |
|  | MSH | HMHA | MSH | HMHA | MSH | HMHA | MSH | HMHA |
| *Women* | *8.70 ± 1.79* | *8.25 ± 2.10* | *6.17 ± 3.12* | *5.58 ± 3.19* | *1.35 ± 2.40* | *1.43 ± 2.38* | *78%* | *68%* |
| 6 to 9 | 7.91 ± 2.65 | 7.77 ± 2.70 | 4.75 ± 3.88 | 3.98 ± 4.02 | 2.46 ± 3.28 | 1.91 ± 2.98 | 67% | 61% |
| 10 to 19 | 8.40 ± 1.92 | 8.25 ± 2.07 | 5.80 ± 3.12 | 5.14 ± 3.15 | 1.26 ± 2.42 | 1.13 ± 2.09 | 74% | 66% |
| 20 to 29 | 8.90 ± 1.60 | 8.39 ± 1.96 | 6.42 ± 2.74 | 5.90 ± 2.92 | 1.41 ± 2.42 | 1.35 ± 2.22 | 81% | 69% |
| 30 to 39 | 8.95 ± 1.49 | 8.32 ± 2.08 | 6.58 ± 2.93 | 5.83 ± 2.92 | 1.21 ± 2.08 | 1.48 ± 2.32 | 79% | 68% |
| 40 to 49 | 9.00 ± 1.48 | 8.42 ± 1.90 | 6.71 ± 2.93 | 6.28 ± 3.04 | 1.01 ± 2.08 | 1.40 ± 2.47 | 84% | 76% |
| 50 to 59 | 8.86 ± 1.87 | 8.34 ± 1.99 | 6.43 ± 3.23 | 6.13 ± 3.16 | 1.34 ± 2.32 | 1.77 ± 2.63 | 79% | 71% |
| 60 to 69 | 8.89 ± 1.24 | 7.91 ± 2.17 | 6.33 ± 3.31 | 5.74 ± 3.29 | 1.55 ± 2.67 | 1.94 ± 2.68 | 84% | 68% |
| 70 to 90 | 7.96 ± 2.07 | 6.88 ± 2.53 | 4.12 ± 3.17 | 3.62 ± 2.87 | 1.31 ± 1.76 | 1.92 ± 2.65 | 73% | 54% |
| *Men* | *8.37 ± 1.93* | *7.98 ± 2.16* | *5.86 ± 3.13* | *5.17 ± 3.21* | *1.88 ± 2.63* | *1.93 ± 2.58* | *74%* | *64%* |
| 6 to 9 | 8.23 ± 2.35 | 8.26 ± 2.45 | 3.93 ± 4.02 | 3.18 ± 3.80 | 2.46 ± 3.44 | 1.70 ± 2.93 | 70% | 62% |
| 10 to 19 | 8.14 ± 2.04 | 7.86 ± 2.22 | 5.23 ± 3.21 | 4.62 ± 3.26 | 2.00 ± 2.82 | 1.86 ± 2.66 | 66% | 63% |
| 20 to 29 | 8.16 ± 2.08 | 7.86 ± 2.20 | 6.52 ± 2.71 | 5.64 ± 2.97 | 1.96 ± 2.56 | 2.16 ± 2.51 | 77% | 66% |
| 30 to 39 | 8.67 ± 1.66 | 8.15 ± 2.05 | 6.41 ± 2.77 | 5.77 ± 2.85 | 1.50 ± 2.18 | 1.74 ± 2.36 | 77% | 61% |
| 40 to 49 | 8.61 ± 1.57 | 7.85 ± 1.95 | 6.82 ± 2.40 | 5.90 ± 2.72 | 1.62 ± 2.13 | 2.02 ± 2.40 | 83% | 65% |
| 50 to 59 | 8.83 ± 1.58 | 8.41 ± 1.87 | 6.17 ± 2.85 | 6.23 ± 2.84 | 1.96 ± 2.87 | 2.14 ± 2.79 | 84% | 71% |
| 60 to 69 | 8.41 ± 1.48 | 7.69 ± 2.41 | 6.93 ± 2.40 | 5.48 ± 3.02 | 2.07 ± 2.51 | 2.24 ± 2.47 | 86% | 76% |
| 70 to 90 | 8.21 ± 2.27 | 7.63 ± 2.54 | 4.58 ± 3.58 | 4.26 ± 3.78 | 1.21 ± 1.51 | 2.16 ± 2.93 | 63% | 58% |
| *All* | *8.58 ± 1.85* | *8.15 ± 2.13* | *6.05 ± 3.13* | *5.42 ± 3.20* | *1.55 ± 2.50* | *1.62 ± 2.47* | *77%* | *67%* |
